# Determination of Colistin in Contents Derived from Gastrointestinal Tract of Feeding Treated Piglet and Broiler

**DOI:** 10.3390/antibiotics10040422

**Published:** 2021-04-12

**Authors:** Chun Peng, Sanling Zuo, Yinsheng Qiu, Shulin Fu, Lijuan Peng

**Affiliations:** 1 School of Animal Science and Nutritional Engineering, Wuhan Polytechnic University, Changqing Garden, Hankou, Wuhan 430023, China; Pengchun1998@163.com (C.P.); Zuosl30@163.com (S.Z.); Qiuyinsheng6405@163.com (Y.Q.); fushulin2016@126.com (S.F.); 2 School of Food Science and Engineering, Wuhan Polytechnic University, Changqing Garden, Hankou, Wuhan 430023, China

**Keywords:** colistin, swine, broiler, gastrointestinal tract, mass spectrometry

## Abstract

Colistin is considered as the last-resort treatment for multiantibiotic-resistant Gram-negative bacterial infections in humans. However, the oral administration of colistin to livestock and poultry results in the introduction of large amounts of colistin to the surrounding environment via urine and feces, potentially inducing the prevalence of colistin-resistant bacteria and the impact on the ecological environment. We established a quantitative mass spectrometry (MS) based method to measure colistin in contents recovered from the gastrointestinal segments of piglets and broilers, as well as colistin in feces from the animals. The mean recoveries of colistin from different matrices were between 73.2% and 103.9%. The quantitation limit values for different matrices ranged from 0.37 to 1.85 ng/g. In colistin-treated swine samples, the highest concentration of colistin was detected in feces samples at a level of 1248.3 ng/g. However, the highest concentration of colistin in broiler samples was around 4882.9 ng/g, which was found in the contents derived from broilers’ ceca. The employment of the proposed method to assess colistin in animals’ gastrointestinal tracts might help to understand the colistin absorption in animals’ guts and the potential impact of colistin on the emergence of resistant bacteria in animals’ gut flora and the ecological environment.

## 1. Introduction

Colistin (polymyxin E) is an old class of cationic polypeptides with broad-spectrum activity against Gram-negative bacteria by disrupting the bacterial membrane to cause cellular death [1,2]. In recent years, many bacterial strains have developed strong resistance to multiple antibiotics, and colistin has been considered as the last-resort treatment for the emergence of multidrug-resistant Gram-negative bacterial infections [3,4,5]. Colistin includes colistin A (polymyxin E1) and colistin B (polymyxin E2), which differ only in their fatty acid tails and have both been widely used as a veterinary medicine for the promotion of growth and prevention and control of diseases in livestock and poultry [6,7]. Currently, two forms of colistin are commercially available, including colistin sulfate for oral and topical use, and sodium colistimethate for parenteral and aerosol therapy. The oral administration of colistin sulfate is employed worldwide in veterinary medicine for the prevention and the treatment of colibacillosis.

However, colistin is poorly absorbed from the gastrointestinal tract [8]. A large amount of the colistin consumed by animals through feed can be excreted in urine and feces into the surrounding environment. The long-term treatment with colistin may result in the accumulation of colistin in animals’ gastrointestinal tracts, and potentially induce the development and prevalence of antimicrobial-resistant bacteria and resistance genes. The tremendous increase in infections caused by antibiotic-resistant bacteria is an ecosystem problem threatening the interrelated human–animal–environment health under the “One Health” framework [9]. The excretion of antimicrobial-resistant bacteria and resistant genes in feces into the ecological environment may spread antibiotic resistance via cross-reservoir transmission to other species or humans, by direct exposure or through the food chain and the environment. Although the prevalence of colistin resistance is currently relatively low worldwide, colistin-resistant isolates have recently been identified in several Gram-negative bacteria species, such as *A. baumannii*, *K. pneumoniae* and *P. aeruginosa* [10]. Extensive use of colistin in the animal industry has led to increased colistin resistance rates [11]. In 2015, the first plasmid-mediated mobile-colistin resistance (*mcr-1*) gene in *Enterobacteriaceae* was discovered in China, and subsequently found in more than 50 countries and regions [7,12,13].

Lately, a positive association between the administration of colistin to swine and the accumulation of *mcr-1*in swine manure samples was discovered [14]. Moreover, colistin-resistant *Escherichia coli* was also observed in wild rabbits (*Oryctolagus cuniculus*) and wild hares (*Lepus europaeus*) without the treatment of colistin [15]. This suggests that colistin-resistant genes might be present in the environment and cause the potential transmission of colistin-resistant *E. coli* between species, particularly from swine [16] or from pets [17] to humans. Based on recent studies of plasmid-mediated colistin resistance *mcr-1* gene, livestock has been considered as the principal reservoir for colistin resistance amplification and spread [8]. Thus, colistin has been banned as a growth promoter (feed additive) in animal production in China since April 2017 [18]. The therapeutic use of colistin in livestock and poultry should be reasonably guided to reduce the prevalence and transmission of colistin resistance.

To regulate the use of colistin, respective maximum residue limits (MRLs) in animal tissues, eggs and milk have been established worldwide. For instance, MRLs of colistin in animals established in China and European communities are 50 ng/mL in milk; 150 ng/g in muscle, liver, and fat; and 200 ng/g in kidneys. To date, extensive work on the development of methods for the determination of colistin in a variety of matrices, including feed, animal tissues, eggs, milk, plasma and feces, has been performed [19,20,21,22,23,24]. However, the determination of colistin in animal tissues, eggs, milk and feces is not sufficient to validate the rationality of the therapeutic use of colistin in animals due to the poor absorption of colistin in the gastrointestinal tracts of animals after oral administration. Changes in colistin concentration in different segments of the animals’ gastrointestinal tracts reflect the absorption and degradation of colistin. Therefore, studies on colistin in the gastrointestinal tracts of animals can assist in the understanding of the potential effects of colistin on the development of colistin-resistant bacteria in animals’ guts and the rational therapeutic use of colistin. So far, scarce data on the concentration and distribution of colistin in animals’ gastrointestinal tracts after oral administration have been reported.

The goals of the study were to establish a robust and sensitive method capable in the determination of colistin in contents derived from different segments of swine and broiler gastrointestinal tracts, using ultrahigh-performance liquid chromatography-tandem mass spectrometry (UPLC–MS/MS), to investigate the concentration and absorption of colistin in different segments of animals’ gastrointestinal tracts for the rational utilization of colistin.

## 2. Results

### 2.1. Method Validation

**Figure 1:**
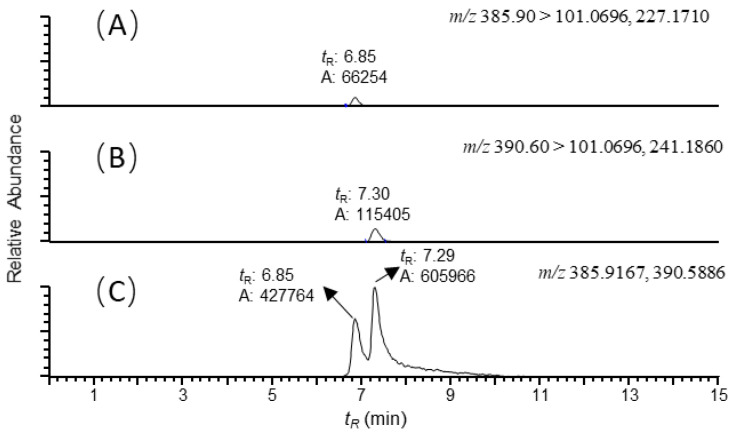
Reconstructed ion chromatograms of (**A**) colistin B ([M+3H]^3+^, *m/z* 385.90 → 101.0696, 227.1710); (**B**) colistin A ([M+3H]^3+^, *m/z* 390.60 → 101.0696, 241.1860); and (**C**) colistin B and A ([M+3H]^3+^, *m/z* 385.9167, 390.5886) recovered from the duodenum sample of swine with colistin at 5ng/g. The scales of the signals were fixed to the maximum response of the analytes. Mass tolerance was set at 5 ppm.

Estimates of colistin in various matrices were determined by using ion signals of selected fragment ions with a mass tolerance of 5 ppm. The corresponding chromatograms of the MS ion signals for blank and untreated samples were quite clear, and no interfering peaks were observed, demonstrating the specificity and reliability of the proposed assay (Figure 1). It was demonstrated that various matrices exhibited significant effects on ion signals for colistin. To evaluate the accuracy of the method, fortified samples were prepared for each matrix at three levels (5, 25 and 50 ng/g) by spiking the colistin standard into 2 g of matrix. Recoveries of colistin in different matrices ranged from 73.2% to 103.9%. The intraday and interday measurements of colistin recoveries in different matrices were determined on three different days over a time period of one week. The within-day coefficient of variation (%) was lower than 6.6%, and between-day coefficient variation was lower than 11.4%. Therefore, five matrix-fortified calibration standards set was employed to construct a linear regression equation for each matrix obtained from different segments of gastrointestinal tract of piglets and cobb broilers, to inherently correct recoveries and compensate for matrix effects. Table 1 and Table 2 show the calibration curve equation, regression coefficient (*r^2^*), limit of detection (LOD) and limit of quantitation (LOQ) of colistin in different matrices. Good linearity was obtained with coefficients >0.994 for colistin in all the matrices. The LODs were in the range of 0.11~0.56 ng/g for colistin in different matrices. The LOQ for colistin in tested matrices was as low as 0.37 ng/g, which was satisfactory for surveillance monitoring. As compared to the data reported previously [14], the LOQ obtained by the proposed method for colistin in swine feces was decreased by more than 10-fold.

### 2.2. Analysis of Swine Samples

**Figure 2:**
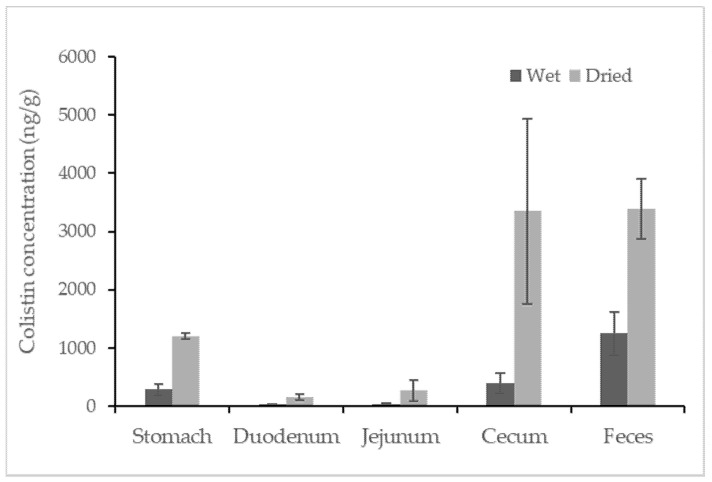
Concentration of colistin in contents recovered from segments of the swine gastrointestinal tracts and feces. The calculation was based on both wet weight (black) and dried weight (grey) of each content (n = 4).

Colistin was found in all samples from colistin-treated piglets, but no colistin was detected in any untreated swine sample. The concentrations of colistin recovered from the different segments of the swine gastrointestinal tracts and feces are reported in Figure 2. Among the tested samples, the average concentration of colistin in feces samples was approximately 1248.3 ng/g, which was statistically higher than that in the content of the gastrointestinal tracts. The detected concentrations of colistin in swine feces samples were consistent with earlier findings in which the concentration of colistin in fresh manure samples collected from five swine farms was reported in the range of below the LOQ to 17,383 ng/g [14]. For content samples from the gastrointestinal tract, the content from the cecum contained relatively high concentrations of colistin at a level of 398.3 ± 161.2 ng/g (mean ± standard deviation). Taking moisture into account, the average amounts of colistin in feces and in content recovered from the cecum were comparably high (~3300 ng/g) when calculations were based on the dried weight of the samples. Compared to the samples mentioned above, samples recovered from the stomach contained a relatively lower level of colistin, followed by samples from the duodenum and jejunum, which contained similar concentrations of colistin at an average level of ~30 ng/g. Data for the ileum sample were not obtained due to insufficient contents for analysis. The trend of colistin distribution in the swine gastrointestinal tracts could be due to the absorption of colistin in swine duodenum and jejunum and the accumulation of colistin in swine ceca.

### 2.3. Analysis of Broiler Samples

**Figure 3:**
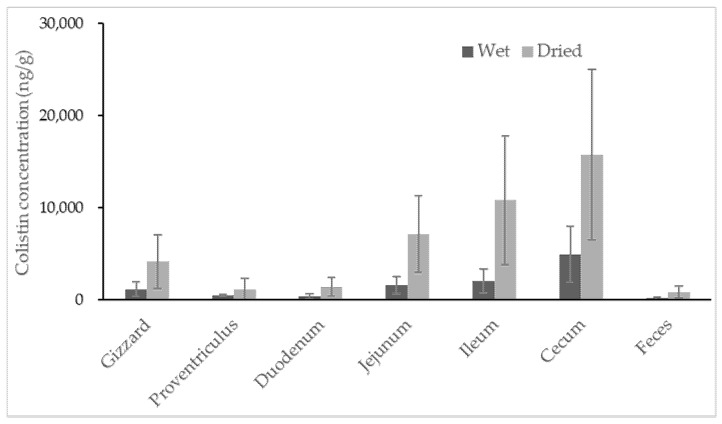
Concentration of colistin in contents recovered from segments of the broiler gastrointestinal tracts and feces. The calculation was based on both wet weight (black) and dried weight (grey) of each content (n = 8).

No colistin was detected in untreated broiler samples. All samples collected from colistin-treated broilers contained colistin, with the highest concentration observed in content recovered from the cecum (Figure 3). Colistin determined in content samples from the cecum ranged from 1348.0 to 11528.6 ng/g, with a high coefficient of variance at 62.9%, indicating significant individual variation among broilers. For content samples recovered from different segments of the broilers’ gastrointestinal tracts, the average concentrations of colistin were 1145.7, 448.3, 383.7, 1572.3, 2056.1 and 4882.9 ng/g for gizzard, proventriculus, duodenum, jejunum, ileum and cecum samples, respectively.

## 3. Discussion

The compositions of contents recovered from different segments of animals’ gastrointestinal tracts and feces are distinct from one another. The matrix effect usually has a significant influence on the recovery of analytes. Moreover, the amount of colistin in different matrices derived from the gastrointestinal tracts of colistin-treated animals may vary considerably due to the absorption and degradation of colistin. Thus, the primary challenges for establishing a universal method to determine colistin in these matrices are appropriate recoveries and sensitivity. In this study, the solid phase extraction (SPE) purification of colistin in different matrices by a weak cation exchange (WCX) cartridge, followed by vacuum centrifugation for dryness, ensured the appropriate recoveries of colistin, especially when analytes were at low concentrations. The employment of high-resolution mass spectrometry combined with ultra-high performance liquid chromatography under optimized conditions offered lower LOQs for colistin in the different analyzed matrices. The LOQ values for colistin in tested matrices ranged from 0.37 to 1.85 ng/g, which were far below the MRLs for the animals’ tissues. This suggests that the presented assay can help to monitor the absorption and degradation of colistin in animals’ gastrointestinal tracts.

**Figure 4:**
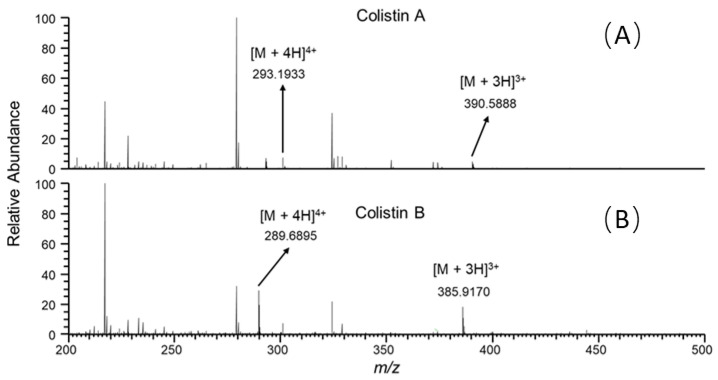
Full scan mass spectra of the duodenum sample from swine with colistin at 5 ng/g, acquired at retention time of (**A**) 7.30 and (**B**) 6.85 min.

It is well known that ion suppression or ion enhancement caused by the sample matrix and interferences from metabolites is always the main issue in the effect of the accuracy and reliability of UPLC–MS/MS-based assays for biological samples [25]. The colistin A and B examined are cationic polypeptides, which generally form multicharged ions under electrospray ionization (ESI). In this study, both colistin A and colistin B appeared as triple and quadruple pronated ions in mass spectra at a *m/z* range < 400, arising with severe interference ions from the matrix, solvent and other contaminants (Figure 4). Quantitative analysis of colistin in different matrices was based on the ion signals of fragment ions of colistin A and B, ensuring the specificity and reliability of the method. However, the fragmentation of parent ions usually leads to decreased ion signals, thus affecting the sensitivity of the method. The extraction of parent ions from full scan mass spectra with a mass tolerance of 5 ppm could eliminate most interference ions (Figure 1C). According to our data, selecting the ion signal of parent ions instead of fragment ions for quantitation could lower the LOQ for each matrix by at least 3-fold. Moreover, lower LOQs may be achieved by assaying more samples, or by the selective enrichment of colistin prior to MS/MS analysis.

Nutrients and drugs are known to be mainly absorbed in the small intestine of animals and humans after oral administration. The dosing regimen of colistin in clinical use depends upon the pharmacokinetics and pharmacodynamics of colistin in humans [26]. Meanwhile, the absorption of oral drugs in the digestive tract is crucial for the dosing regimen as well. Unlike for human medicine, a few studies have been conducted in animals to evaluate the pharmacokinetics and pharmacodynamics of colistin following oral administration [8,27,28]. One study has measured colistin in the whole intestinal tracts (from the duodenum to ileum) of pigs after a single oral administration, using high-performance liquid chromatography assays (HPLC-Fluorescence). Since colistin cannot be readily detected by HPLC detectors, the derivatization of colistin was conducted prior to HPLC analysis. Peak concentrations of colistin in samples from the duodenum to ileum were achieved rapidly after administration, at the highest level of ~90,000 ng/g. However, no colistin was detected in any of the samples from duodenum to ileum after 4 h [28]. According to our data, this was likely due to the poor sensitivity of the HPLC assay. Our study provided the trend of colistin distribution in the swine gastrointestinal tract, indicating that the absorption of colistin probably occurred in the swine duodenum and jejunum. The slightly higher amount of colistin found in contents recovered from the swine jejunum might be attributed to less absorption of colistin in the jejunum than in the duodenum of swine. The accumulation and lower absorption of colistin might contribute to significantly increased amounts of colistin in the contents of swine ceca. As polypeptides, colistin may be degraded by proteolytic enzymes and microorganisms in the swine gastrointestinal tract. Differences in the cecal microbiota of individuals reportedly occur in swine [29], suggesting that the degradation of colistin in the swine cecum may differ in individuals. This may explain the observed high variability of colistin in the cecum samples of swine.

The two lowest concentrations of colistin were discovered in content samples from a broiler’s proventriculus and duodenum, which were probably the primary location of colistin absorption. The average concentrations of colistin gradually increased in the content of the jejunum, ileum and cecum, suggesting less or no absorption of colistin in these areas. It was found that colistin recovered from all segments of the gastrointestinal tract was highly varied in individuals. The specific dominant bacterial community was reported in every broiler, as well as every compartment of each broiler’s intestinal tract [30,31]. Thus, the degradation of colistin caused by bacteria in each segment of broiler intestinal tract may be different from one another, resulting in high variabilities of colistin recovered from each segment of intestinal tract among individuals. It is noteworthy that the feces samples of broilers had very little colistin, with an average concentration of 147.7 ng/g. Regardless of the moisture, the estimates of colistin in samples from the cecum and feces (mean ± standard deviation) were 15766.0 ± 9249.2 and 823.4 ± 616.4 ng/g, respectively. The amount of colistin significantly decreased from the cecum to feces by at least 5-fold. The rectum of broilers is short; colistin can usually be excreted quickly from the broiler’s cecum to the surrounding environment. The disappearance of most colistin during transferring from the cecum to feces could not be caused by the absorption of colistin in the large intestine of broilers, but possibly by the degradation of colistin in the large intestine. Unfortunately, the concentration of colistin in content samples from the large intestine was unavailable due to insufficient samples for analysis. Studies are ongoing to reveal the potential metabolisms of colistin in broilers’ large intestines.

The presented data signify that colistin used in veterinary medicine by oral administration leads to elevated colistin in the contents of animals’ gastrointestinal tracts and feces, and thereby explains the importance and urgency of the judicious therapeutic use of colistin in animals.

## 4. Materials and Methods

### 4.1. Chemicals and Materials

Colistin sulfate (colistin A and colistin B mixture) was purchased from the National Institute of Control of Pharmaceutical and Biological Products (Beijing, China). LC-MS grade methanol, acetonitrile, water and formic acid (50%) were obtained from Thermo Fisher Scientific (San Jose, CA, USA). Oasis WCX SPE cartridges (3 cc/60 mg) were from Waters (Milford, MA, USA). All other reagents were analytical grade and purchased from Sinopharm Chemical Reagent (Shanghai, China).

### 4.2. Animal Treatment

Eight weaned piglets (Duroc × Landrace × Large white) 3~4 weeks of age were used in a 14-day feeding trial. First, all piglets were fed with the base diet (Table S1) for an adapting period of 6 days. Subsequently, four piglets fed the base diet were kept as untreated controls, and the other four piglets were fed the base diet, which was supplemented with colistin sulphate at 20 mg/kg of body weight for 14 consecutive days. Thereafter, feces were collected, and the piglets were slaughtered to obtain contents from the swine gastrointestinal segments, including stomach, duodenum, jejunum and cecum. The sixteen cobb broilers 1–3 days in age were selected and fed with a drug-free balanced diet (Table S2) during the 14-day adaption period and the subsequent treatment period. Then, eight broilers were kept as untreated controls, and the other eight broilers were fed with colistin at 20 mg/kg of body weight for 14 consecutive days. At the end of the trial, treated and untreated animals were sacrificed, and the contents derived from the gizzard, proventriculus, duodenum, jejunum, ileum and cecum were recovered. All samples were stored at −80 °C before being analyzed.

### 4.3. Extraction of Colistin from Contents and Feces

The extraction procedure was based on the methods developed by Fu [20] with some modifications. Briefly, 2 g of samples was lyophilized to dryness and extracted twice with 10 mL of 10% trichloroacetic acid: acetonitrile (40:60, *v/v*), and the combined extraction solution was adjusted to pH 9.0. Thereafter, the extraction solution was applied to Oasis WCX SPE cartridges which were prewashed with 3 mL of methanol and 3 mL of water. The cartridges were then rinsed with 3 mL of water, 3 mL of methanol and 3 mL of 5% formic acid in water. Eventually, the colistin was eluted with 5 mL of 20% formic acid in methanol. The eluent was evaporated to dryness by vacuum centrifugation at 50 °C and resuspended in 500 μL of deionized water prior to measurement using an UPLC–MS/MS system.

### 4.4. UPLC-MS/MS

Chromatography of colistin was conducted with an UltiMate 3000 UPLC system (Thermo Fisher, San Jose, CA, USA) equipped with an Acquity UPLC BEH C18 column (2.1 mm × 100 mm; 1.7 µm particle size; Waters, Milford, MA, USA). The mobile phase A was 0.2% formic acid, 5% acetonitrile in H_2_O; B was 100% acetonitrile. The colistin was resolved with a linear gradient starting from 95% A solvent to 70% B solvent over 5 min and then to 5% A at 10 min, at a flow rate of 200 µL/min.

MS spectra were acquired at positive ionization mode with a hybrid quadrupole-obitrap mass spectrometer (Q Exactive, Thermo Fisher, San Jose, CA, USA). The sheath gas flow rate was 25 mL/min; the auxiliary gas flow rate was 15 mL/min; the spray voltage was 3.2 kV; and the temperature of the capillary tube was set to 320 °C. One microscan was used for data acquisition. The resolution settings were full MS target 70,000 and MS/MS target 17,500; normalized CE 20% and 22% were employed for colistin A and colistin B, respectively. Data manipulations were employed using Xcalibur version 4.0 software. Quantitative analyses were conducted in the selective reaction monitoring (SRM) scan mode. The two most abundant fragment ions were selected from each analyte for analysis (Figure S1). The transitions monitored and approximate chromatographic retention times (*t*_R_) were as follows: colistin A ([M + 3H]^3+^), *m/z* 390.60 > 101.0696, 241.1860, *t_R_* = 7.30 min; colistin B ([M + 3H]^3+^), *m/z* 385.90 > 101.0696, 227.1710, *t_R_* = 6.85 min. The mass tolerance was set to 5 ppm for fragment ions.

### 4.5. Method Validation

Estimates of colistin were determined by the total signals of selected fragment ions for colistin A and B. The method was validated in terms of specificity, recovery, accuracy, precision and linearity. The specificity of the method was investigated by checking potential ion signals of interferences from blank and untreated animal samples. The accuracy and recovery of the method was evaluated by recovery experiments at 3 fortified concentrations of colistin (5, 25, 50 ng/g) in contents derived from different segments of gastrointestinal tract of piglets and broilers. Samples were prepared in triplicate. The precision was determined by intraday and interday assay. The LOD and LOQ values of colistin in different matrices were calculated by the signal-to-noise ratio of 3 (S/N = 3) and 10 (S/N = 10), respectively.

### 4.6. UPLC–MS/MS Analysis for Real Samples

Colistin-treated samples and controls were subject to analysis using the proposed method, including stomach, duodenum, jejunum and cecum content samples and feces samples recovered from 4 treated and 4 untreated piglets; gizzard, proventriculus, duodenum, jejunum, ileum and cecum content samples and feces samples from 8 treated and 8 control cobb broilers. Five-point matrix-matched calibration standard curves, over a concentration range covering the concentrations expected in animal samples, were prepared with each content sample from the gastrointestinal segments and feces samples recovered from untreated animals. Confirmation criteria were that the selected fragment ions in the samples were within the tolerance range of 5 ppm in matrix-matched standards, and the retention time of the compound in the samples within ±2% of the retention time of the matrix-matched standards.

## 5. Conclusions

Currently, the oral administration of colistin is still among the main means for the treatment of infections caused by multidrug resistance in animal husbandry. A large amount of colistin residue and potential colistin-resistant bacteria can be excreted into the surrounding environment via animals’ feces, which affects environmental microbes, causing an impact on the prevalence of colistin resistance in humans. However, extensive work has focused on the pharmacokinetics and pharmacodynamics of colistin, while scarce research has been carried out on the absorption of colistin in animals’ gastrointestinal tracts. This is the first time that an MS-based assay has been developed to measure colistin in contents obtained from different segments of gastrointestinal tract of swine and broilers. The proposed method is demonstrated to be accurate and robust. The LOQs of colistin in the matrices tested were as low as 0.37 ng/g. Colistin was detected in all colistin-treated samples. For possible samples with a concentration below LODs, lower LOQs may be achieved by assaying more samples, or by the selective enrichment of colistin prior to MS/MS analysis. The employment of the proposed method to assess the concentration of colistin in contents recovered from different segments of animals’ gastrointestinal tracts will help to understand the colistin absorption in animals’ guts, and the potential impact on animals’ gut flora and the ecological environment. Furthermore, the validated method may assist in monitoring colistin in the animal treatment to ensure a judicious therapeutic use of colistin in animals.

## Figures and Tables

**Table 1:** Calibration curve equation, *r*^2^, LOD and LOQ values for colistin in each matrix from the swine gastrointestinal tracts and feces.

Gastrointestinal Segment/Feces	Linear Equation	*r*^2^ Values	LOD (ng/g)	LOQ (ng/g)
Stomach	y = 39926x − 168707	0.9992	0.28	0.93
Duodenum	y = 50445x − 521015	0.9968	0.29	0.96
Jejunum	y = 44455x − 114407	0.9974	0.13	0.45
Ileum	y = 42583x − 234343	0.9964	0.13	0.42
Cecum	y = 40806x − 160046	0.9948	0.32	1.08
Feces	y = 51895x − 307464	0.9997	0.28	0.93

**Table 2:** Calibration curve equation, *r*^2^, LOD and LOQ values for colistin in each matrix from the broiler gastrointestinal tracts and feces.

Gastrointestinal Segment/Feces	Linear Equation	*r*^2^ Values	LOD (ng/g)	LOQ (ng/g)
Gizzard	y = 35126x + 539167	0.9990	0.26	0.86
Proventriculus	y = 41242x − 87577	0.9965	0.17	0.57
Duodenum	y = 41551x + 215899	0.9962	0.11	0.37
Jejunum	y = 38034x + 134109	0.9958	0.42	1.39
Ileum	y = 38529x − 155306	0.9944	0.27	0.89
Cecum	y = 42291x + 260184	0.9970	0.23	0.78
Feces	y = 42184x + 231232	0.9966	0.56	1.85

## Data Availability

Data is contained within the article.

## Supplementary Materials

The following are available online at https://www.mdpi.com/article/10.3390/antibiotics10040422/s1, Table S1: Diet composition for swine as fed; Table S2: Diet composition for broiler as fed; Figure S1: Product ion mass spectra of (A) colistin B ([M+3H]^3+^at m/z 385.90) and (B) colistin A ([M+3H]^3+^at m/z 390.60).
