# Supplementary material for: Determination of Colistin in Contents Derived from Gastrointestinal Tract of Feeding Treated Piglet and Broiler
Source: Antibiotics (Basel). 2021 Apr 12;10(4):422. doi: 10.3390/antibiotics10040422 (PMC8070394; doi:10.3390/antibiotics10040422)
Supplement: Supplementary file 1 [file antibiotics-10-00422-s001.pdf]

Supplementary Materials

Determination of Colistin in Contents Derived from Gastrointestinal Tract of Feeding Treated Piglet and Broiler

Chun Peng [1], Sanling Zuo [1], Yinsheng Qiu [1], Shulin Fu [1] and Lijuan Peng [2,*]

[1] School of Animal Science, Wuhan Polytechnic University, ChangQing Garden, Hankou, Wuhan 430023, China; Pengchun1998@163.com (C.P.); Zuosl30@163.com (S.Z.); Qiuyinsheng6405@163.com (Y.Q.); fushulin2016@126.com (S.F.)
[2] School of Food Science and Engineering, Wuhan Polytechnic University, ChangQing Garden, Hankou, Wuhan 430023, China
* Correspondence: lijuan_peng@hotmail.com; Tel.: +86-278-395-6442

Table S1. Diet composition for swine as fed.

**Component**	**Content (%)**
Corn	63.52
Soybean meal	22
Concentrated soybean	3.85
Fermenting soybean powder	3.45
Whey powder	3.65
Glucose	3.53

Table S2. Diet composition for broiler as fed.

**Component**	**Content (%)**
Corn	51.73
Soybean meal	40.73
Soybean oil	3.36
Calcium monophosphate	1.92
Limestone	1.16
salt	0.35
DL-methionine	0.26
Choline chloride (50%)	0.25
Mineral complex	0.2
Vitamin complex	0.04

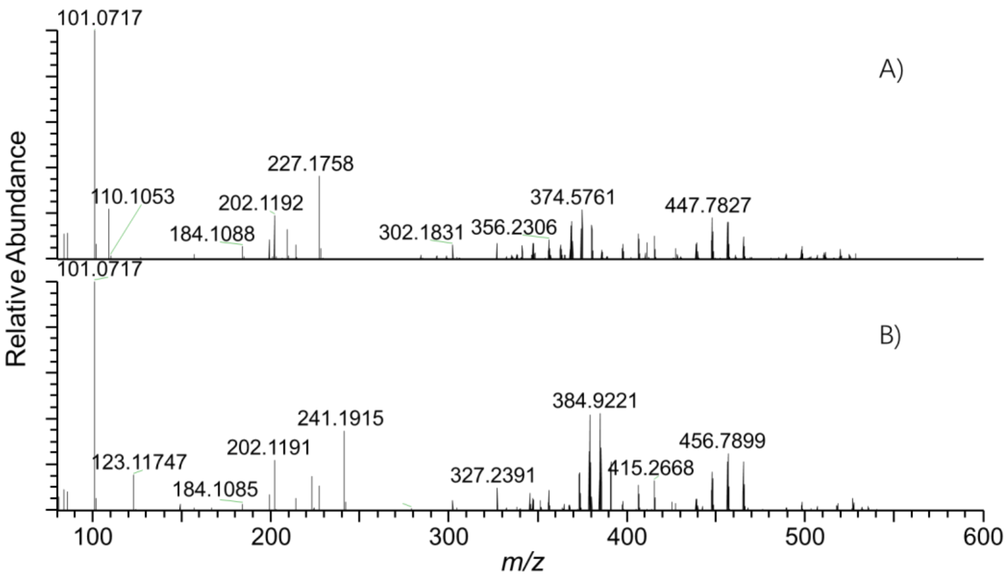

Figure S1. Product ion mass spectra of A) colistin B ($[M+3H]^{3+}$at *m/z* 385.90) and B) colistin A ($[M+3H]^{3+}$at *m/z* 390.60).
